# Associations of Social Determinants of Health Scores with Visual Impairment and Premature Death in Visually Impaired Adults: Insights from CHARLS—A Cohort Study

**DOI:** 10.3390/healthcare14152329

**Published:** 2026-08-01

**Authors:** Haodi Xiao, Xinyi Liu, Lijuan Deng, Yunqing Deng, Jun Jiang, Binbin Su

**Affiliations:** National Clinical Research Center for Ocular Diseases, Eye Hospital, Wenzhou Medical University, 270 Xueyuan Road, Wenzhou 325003, China; xiaohaodi@wmu.edu.cn (H.X.); xinyiliu@wmu.edu.cn (X.L.); lijuandeng@wmu.edu.cn (L.D.); yunqingdeng@wmu.edu.cn (Y.D.)

**Keywords:** visual impairment, social determinants of health, premature death, CHARLS

## Abstract

**Highlights:**

**What are the main findings?**

- Higher social determinants of health scores are associated with a lower risk of visual impairment in Chinese adults aged ≥ 45 years.
- Among individuals with visual impairment, higher social determinants of health scores are associated with a significantly lower risk of premature death.

**What are the implications of the main findings?**

- Integrating the social determinants of health-based risk stratification into community eye health screening may help identify older adults at higher risk of visual impairment, facilitating earlier intervention.
- Improving social determinants was associated with lower odds of visual impairment and extended life expectancy in visually impaired populations.

**Abstract:**

Background: The prevalence of vision impairment (VI) has been increasing worldwide. However, limited population-based evidence currently exists on the relationship between VI and social determinants of health (SDH) scores in China. Even less is known about the association between SDH scores and early death, especially in people with VI. This study aimed to evaluate the association between SDH scores and VI, and further examine whether SDH scores were associated with premature mortality among middle-aged and older Chinese adults with VI. Methods: Data from the China Health and Retirement Longitudinal Study (CHARLS) were analyzed in this study. We examined the association between SDH scores and VI among 2559 adults aged ≥ 45 years, and further assessed the relationship between SDH scores and premature death in 8612 individuals aged ≥ 45 years with VI. Statistical analyses included Kaplan–Meier curves, Cox proportional hazards models, restricted cubic spline (RCS) analyses, subgroup analyses, and sensitivity analyses. Results: Kaplan–Meier analysis revealed that individuals with higher SDH scores had a lower risk of experiencing VI compared to those with lower SDH scores (log-rank test *p* < 0.001). Similarly, among individuals with VI, higher SDH scores were consistently associated with a reduced probability of premature death (log-rank test *p* < 0.001). In the fully adjusted model, each unit increment in SDH score was associated with a 6% lower risk of VI (HR = 0.94, 95% CI: 0.91–0.98, *p* = 0.002) and a 15% lower risk of premature death (HR = 0.85, 95% CI: 0.78–0.93, *p* < 0.001). Conclusions: Among Chinese adults aged ≥45 years, higher SDH scores were significantly correlated with a lower risk of VI. Furthermore, low SDH scores were associated with an increased risk of premature death in those with VI. These findings highlight the association between SDH and lower VI risk as well as longer life expectancy for individuals already living with VI among middle-aged and older populations.

## 1. Introduction

Visual impairment (VI) is a major global public health challenge. In 2020, the Global Burden of Disease Study estimated that more than 1.1 billion individuals experienced some form of VI, including 43.3 million who were blind, 295 million with moderate to severe impairment, 258 million with mild impairment, and 510 million with uncorrected presbyopia [1]. Driven by global population aging, the overall burden of VI is projected to continue growing. By 2050, the number of people living with moderate to severe VI is expected to reach 474 million, which will further strain already overburdened healthcare systems [1].

Social determinants of health (SDH) encompass the structural and contextual factors that shape individuals’ living environments and life experiences across the lifespan and predict various health-threatening conditions [2]. In recent years, the association between SDH and hip fractures as well as chronic kidney disease has been reported [3,4]. Moreover, studies have also linked SDH to ocular diseases such as keratopathy and glaucoma, which often lead to VI [5,6]. However, evidence regarding the link between SDH and VI in the Chinese population remains scarce.

Compared with the general adult population, individuals with VI have a higher mortality risk, which increases progressively with the severity of vision loss [7]. First, retinal artery occlusion and other VI conditions raise cardiovascular risk and subsequent mortality [8]. Secondly, individuals with VI have been shown to face a higher risk of suicide, driven by factors such as depression and diminished quality of life [9,10,11]. Furthermore, research from the United States among a nationally representative sample of adults has shown that adverse SDH predict a greater risk of premature death [12]. However, the association between SDH and premature death in Chinese people with VI has yet to be examined.

Using data from the China Health and Retirement Longitudinal Study (CHARLS), a large-scale and nationally representative cohort, this research aims to generate actionable evidence. The core objectives are to evaluate how SDH scores relate to VI in Chinese adults aged ≥ 45 years and to explore SDH’s association with premature death among those with VI. Ultimately, this work seeks to guide policy by demonstrating the necessity of incorporating SDH into strategies for eye health and geriatric care.

## 2. Materials and Methods

### 2.1. Study Design and Population

CHARLS data were used in this investigation. Chinese adults aged ≥45 years made up the CHARLS population [13]. In 2011, a baseline survey was carried out using multistage probability-proportional-to-size sampling, ultimately enrolling 17708 participants from diverse geographic regions across China. The Peking University Ethics Review Committee granted ethical permission for this work (IRB00001052-11015). All participants provided written informed consent, and the data are publicly accessible at http://charls.pku.edu.cn/ (accessed on 4 February 2026). We performed two separate analyses: one evaluating the relationship of SDH scores to VI, and the other assessing prognosis in individuals with VI.

### 2.2. Assessment of SDH

SDH assessment followed the frameworks of WHO and Healthy People 2030 [2,14]. The Chinese version of the SDH scoring system in the CHARLS database selected 10 socioeconomic and health indicators closely related to individual well-being for a comprehensive score. The specific scoring criteria were as follows: Homeowners received 1 point, while renters received 0 points. Household income was dichotomized using a threshold of 9000 yuan per capita per year; households meeting or exceeding this threshold received 1 point, whereas those falling short received 0. Employment status was scored as 1 for employed or retired individuals and 0 for those who were unemployed. Individuals with government-subsidized, private, or self-purchased health insurance received 1 point, while those without insurance or relying on government assistance received 0 points. Regarding educational attainment, a bachelor’s degree or higher received 1 point, while 0 points were assigned to those with lower educational levels. Individuals without transportation barriers to medical care received 1 point, while those facing such barriers received 0 points. Individuals not living alone received 1 point, while those living alone received 0 points. Individuals with a CES-D depression scale score below 10 (indicating no significant depressive symptoms) received 1 point, while those scoring above 10 received 0 points. Regarding social participation, 1 point was awarded for regular participation in social activities, and 0 points for irregular participation in various social activities. Married individuals or those with a stable partner received 1 point, while those who were separated, divorced, widowed, or never married received 0 points. The detailed SDH scoring table is provided in Supplementary Table S1 [3]. Within the CHARLS database, SDH scores spanned from 0 to 10, where higher values reflected a more favorable lifestyle profile. Following a prior study, we classified participants in both analyses into two groups based on their SDH score (≥7 vs. <7) to explore long-term health differences across SDH categories [3].

### 2.3. Assessment of VI

In the CHARLS database, VI was categorized into distance VI and near VI. Both types of VI were assessed using a questionnaire. Participants were asked: “How well can you see objects at a distance, such as recognizing a friend across the street (if wearing glasses or corrective lenses)?” for distance VI, and “How well can you see objects at close range, such as reading a newspaper (if wearing glasses or corrective lenses)?” for near VI. Participants’ responses included: poor, fair, good, very good, excellent. Participants who responded “poor” to either question were defined as having VI.

### 2.4. Assessment of Premature Death

Based on previous research, premature death was defined in this study as death before the age of 70 [15].

### 2.5. Covariates

We adjusted for a range of covariates, including age, sex, body mass index (BMI), smoking/drinking status (never, former or current), educational level (illiterate, incomplete primary school, primary school, junior high school, high school, college or above) and health and pension insurance status (yes/no). Regarding chronic diseases, we considered hypertension, heart disease, diabetes, stroke and dyslipidemia. Standard diagnostic definitions were applied. For hypertension, the criteria included a blood pressure reading of ≥140/90 mmHg, physician-diagnosed hypertension, or treatment with antihypertensive medication. For diabetes, the criteria included an HbA1c level of >6.5%, fasting or random glucose levels of ≥7.0 or ≥11.1 mmol/L, respectively, a 2-h OGTT glucose level of ≥11.1 mmol/L, physician-diagnosed diabetes, or treatment with antidiabetic medication. Dyslipidemia was identified based on any of the following criteria: triglycerides > 150 mg/dL, HDL cholesterol < 40 mg/dL, LDL cholesterol > 160 mg/dL, total cholesterol > 240 mg/dL, or a physician’s diagnosis or treatment. Finally, stroke and heart disease were confirmed based solely on a physician’s diagnosis.

### 2.6. Statistical Analysis

Missing data for all covariates were below 20%. For quantitative data, missing values were imputed using the predicted mean matching method, while for categorical data, logistic regression was used for imputation. Supplementary Table S2 provides details on missing data.

Participants were assigned to two groups based on their baseline SDH scores, using a cutoff of 7 (≥7 vs. <7). For continuous variables, data are presented as mean ± SD and were compared between groups using independent-samples *t*-tests. Categorical variables are shown as n (%) and were analyzed using chi-square tests. The follow-up period started at the 2011 baseline assessment and lasted until the earliest of the following events: first self-reported VI, premature death, loss to follow-up, study completion (in 2018), or other events.

Kaplan–Meier curves and Cox proportional hazards regression were applied to evaluate the association of SDH scores with two outcomes. SDH scores were treated as both continuous and categorical (≥7 vs. <7) variables in separate Cox models. Four models with sequential adjustments were constructed. GVIFs were all < 3, indicating no multicollinearity issues (Supplementary Table S3). The proportional hazards assumption was met (Schoenfeld global test, *p* > 0.05; Supplementary Table S4). To evaluate dose-response relationships, restricted cubic splines (RCS) were fitted with four knots for the VI analysis (owing to the smaller sample size) and five knots for the premature death analysis (owing to the larger sample size).

Subgroup analyses stratified by age, sex, BMI, smoking/drinking status, and five chronic conditions (hypertension, heart disease, dyslipidemia, diabetes, and stroke) were conducted. Likelihood ratio tests assessed SDH score–covariate interactions for each stratification factor. Three sensitivity analyses tested result robustness: (1) Cox regression models were refitted using pre-imputation data; (2) Model 4 was further adjusted for three additional SDH-related covariates: pension insurance, health insurance, and education level; (3) to address potential reverse causality, deaths occurring during the initial two years of follow-up were omitted from the analysis of premature death among individuals with VI.

RStudio 4.4.2 was used for all analyses, and statistical significance was defined as two-sided *p* < 0.05.

## 3. Results

### 3.1. Participant Selection

**Figure 1:**
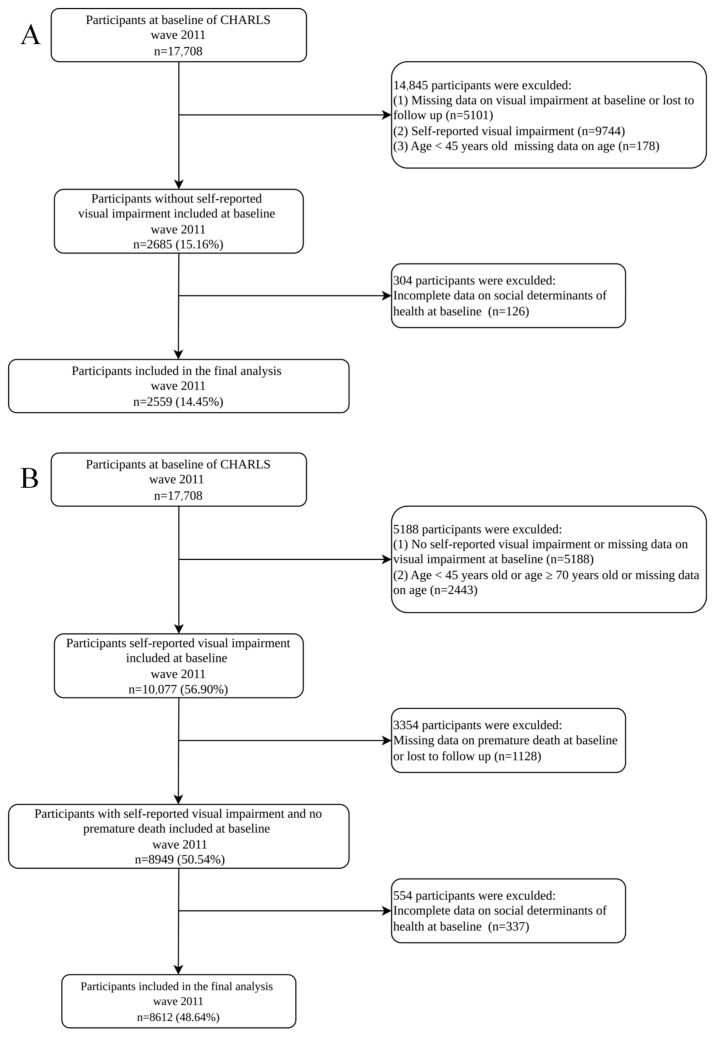
Participant screening and inclusion procedure. (**A**) Selection process for the relationship between social determinants of health scores and VI probability in the general adult population. Of the 17,708 participants at baseline, 2559 were included in the final analysis (retention rate: 14.45%). (**B**) Selection process for the relationship between social determinants of health scores and premature death probability in individuals with visual impairment. Of the 17,708 participants at baseline, 8612 were included in the final analysis (retention rate: 48.64%).

Two analyses were conducted: one evaluating the relationship of SDH scores to VI, and the other assessing prognosis in individuals with VI. Both studies were based on 17,708 participants from the CHARLS baseline survey. However, due to the absence of 2020 VI data in the CHARLS database, the follow-up period for both studies ended in 2018. The first analysis examined the relationship between SDH scores and VI risk in general adult populations. We excluded participants from the final analysis who met any of the following criteria: missing VI data at baseline, were lost to follow-up, self-reported VI at baseline, had incomplete SDH data at baseline, or were aged under 45 years. Following these exclusions, the final analytic sample consisted of 2559 participants (retention rate: 14.5%). Another analysis examined the relationship between SDH scores and premature death risk among individuals with VI. The selection process for the final analysis population was as follows: Of the 12,520 participants who self-reported VI at baseline, we excluded those with missing data on premature death (either at baseline or during follow-up), those with incomplete SDH data at baseline, and those aged under 45 or over 70. Following these exclusions, the final analytic sample consisted of 8612 participants (retention rate: 68.8%). Figure 1 illustrates the detailed participant selection procedures for both analyses.

### 3.2. Participant Characteristics

Table 1 summarizes the baseline characteristics of the study sample for the analysis of VI risk among general adults, stratified by SDH scores (≥7 vs. <7); 558 participants fell into the lower-scores group and 2001 into the higher-scores group. The overall mean age was 56.99 ± 8.97 years. Participants in the higher SDH score group were significantly younger than those in the lower score group (56.17 ± 8.55 vs. 59.92 ± 9.81 years; *p* < 0.001). The study sample comprised 53.03% men and 46.97% women, with men overrepresented in the higher-scores group (56.32% vs. 41.22%; *p* < 0.001). Additionally, significant between-group differences were observed for BMI, smoking status, drinking status, heart disease, and stroke (all *p* < 0.05).

Table 2 summarizes the baseline characteristics of the study sample for the analysis of premature death risk among individuals with VI, stratified by SDH scores (≥7 vs. <7); 2813 participants were assigned to the lower-scores group and 5779 to the higher-scores group. The overall mean age of the participants was 56.72 ± 6.59 years. Those in the higher SDH scores group were significantly younger than their counterparts in the lower-scores group (58.10 ± 6.54 vs. 56.06 ± 6.47 years; *p* < 0.001). The study sample comprised 45.36% men and 54.64% women, with men constituting a greater proportion of the higher-score group (47.96% vs. 39.99%; *p* < 0.001). Additionally, significant between-group differences were observed for BMI, heart disease, stroke, hypertension, smoking status, and drinking status (all *p* < 0.05).

### 3.3. Kaplan–Meier Survival Curve

**Figure 2:**
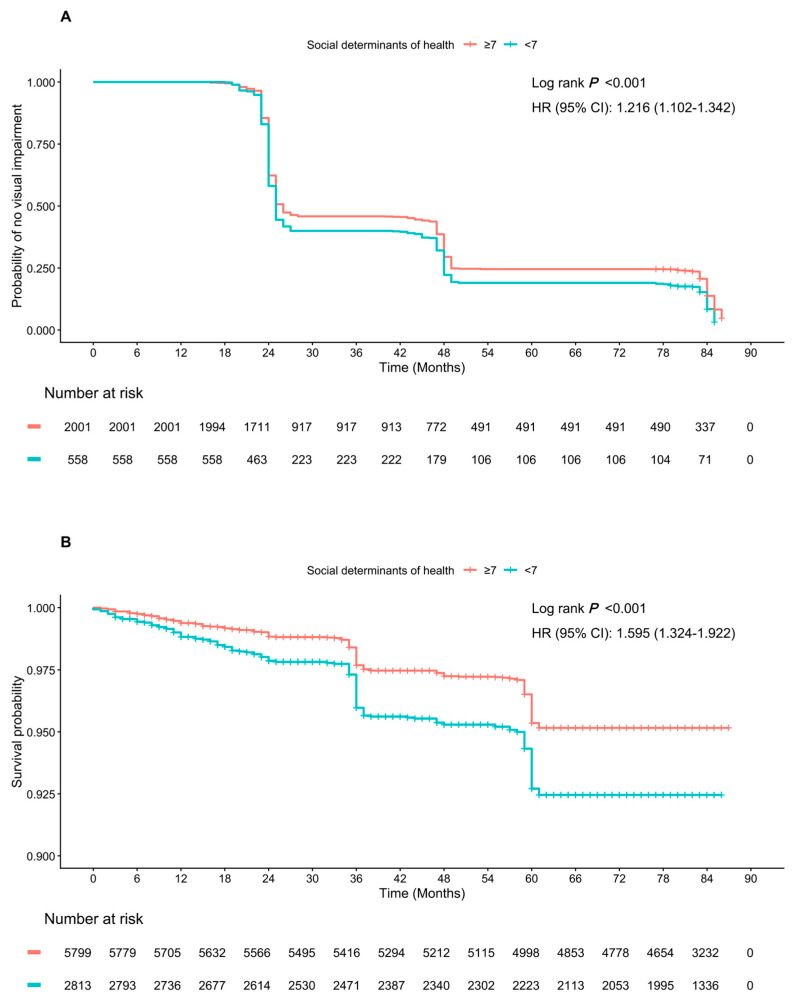
Kaplan–Meier survival curves for SDH scores. (**A**) The risk of visual impairment among the general adults. (**B**) The risk of premature death in individuals with visual impairment.

Figure 2 presents the Kaplan–Meier survival curves, with the population stratified by SDH scores. Figure 2A shows that in the general adult population, participants with higher SDH scores had a consistently lower probability of developing VI than those with lower SDH scores (log-rank test *p* < 0.001). Figure 2B shows that among the individuals with VI, those with higher SDH scores had a consistently lower probability of premature death than those with lower SDH scores (log-rank test *p* < 0.001).

### 3.4. Cox Regression Analyses of SDH Scores and Two Outcomes

SDH scores as a continuous measure. In unadjusted models, higher SDH scores were associated with lower risks of both outcomes. After full adjustment (Model 4), each one-unit increase corresponded to a 6% reduction in VI risk (HR = 0.94, 95% CI: 0.91–0.98, *p* = 0.002) and a 15% reduction in premature death risk among individuals with VI (HR = 0.85, 95% CI: 0.78–0.93, *p* < 0.001) (Table 3). We also estimated absolute risk differences. In the fully adjusted model, higher SDH scores corresponded to an absolute risk reduction of 5.2% for VI and 2.3% for premature death (Supplementary Table S8).

### 3.5. RCS of SDH Scores and Two Outcomes

**Figure 3:**
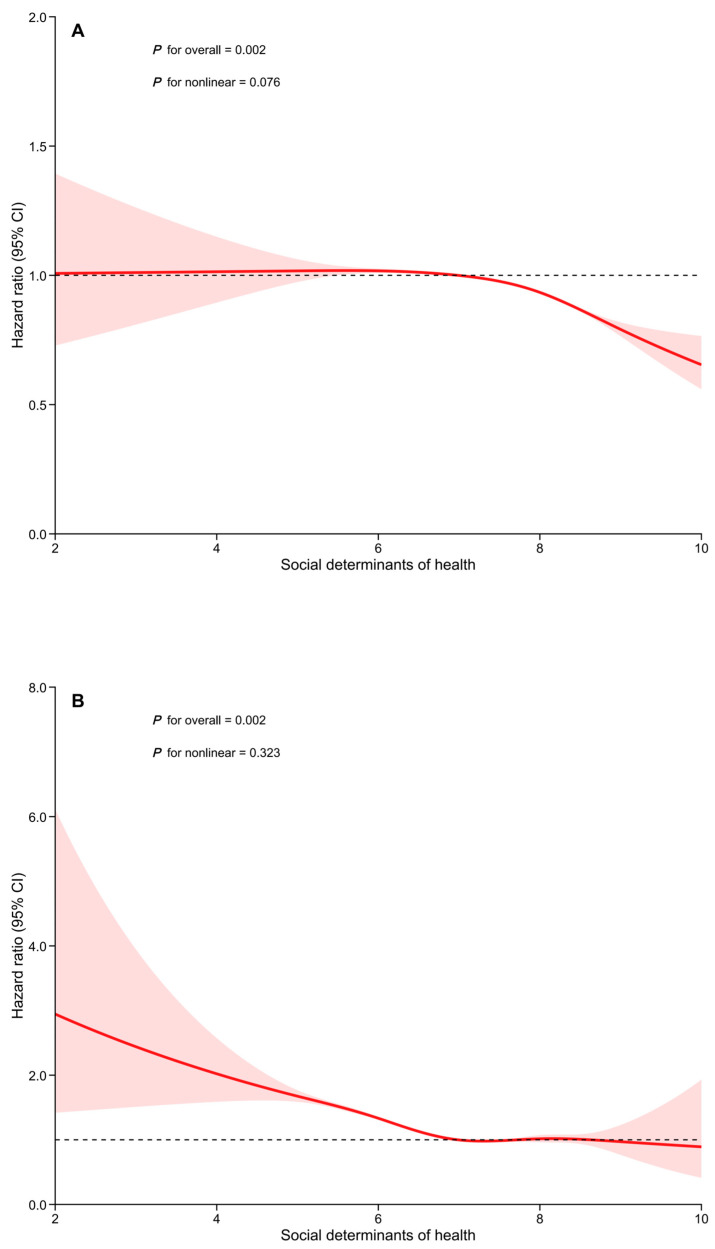
Restricted cubic spline (RCS) analyses. Multivariable-adjusted RCS models were used to examine how social determinants of health scores associate with two outcomes: (**A**) Visual impairment probability in the general adult population. (**B**) Premature death probability among individuals with visual impairment. All models accounted for age, sex, BMI, smoking status, drinking status, heart disease, stroke, diabetes, hypertension, and dyslipidemia.

Covariate-adjusted RCS analyses demonstrated a linear gradient of SDH scores with both outcomes (Figure 3). In the general adult population, higher SDH scores corresponded linearly to a reduced risk of VI (overall *p* = 0.002; nonlinear *p* = 0.076). Likewise, among individuals with VI, higher SDH scores demonstrated a linear relationship with reduced risk of premature death (overall *p* = 0.002; nonlinear *p* = 0.323).

### 3.6. Subgroup and Sensitivity Analyses of SDH Scores and Two Outcomes

To evaluate the robustness of the primary findings, we examined the associations of continuous and categorical SDH scores (≥7 vs. <7) with the outcomes using subgroup analyses across both analytical cohorts (Supplementary Figure S1).

Subgroup analyses were generally in agreement with the primary findings (Table 3). For the association between SDH scores and VI in the general adult population, the negative relationship persisted across most subgroups, regardless of whether SDH scores were modeled continuously or categorically (Supplementary Figure S1A,B). Notably, when SDH scores were modeled as a continuous variable, a significant interaction with BMI was observed (*p* = 0.049). Similarly, when SDH scores were modeled as a categorical variable, a significant interaction with stroke was observed (*p* = 0.037). However, the former interaction was not observed for categorical SDH scores (*p* = 0.054), and the latter was not observed for continuous SDH scores (*p* = 0.380). For the association between SDH scores and premature death among individuals with VI, the inverse relationship remained robust across most subgroups for both continuous and categorical SDH scores (Supplementary Figure S1C,D). A significant interaction was observed between hypertension and SDH scores, irrespective of whether SDH scores were modeled as continuous or categorical (all *p* < 0.01).

In the sensitivity analyses, both studies performed Cox regression analyses using pre-imputation data, and the results remained materially unchanged (Supplementary Table S5). Similarly, in both studies, three SDH-related variables: pension insurance, health insurance, and educational level—were included as covariates in Model 4 for Cox regression analysis. The results remained materially unchanged (Supplementary Table S6). In the analysis of the association between SDH scores and premature death among individuals with visual impairment, a Cox regression analysis was conducted after removing deaths recorded within the initial 2-year follow-up period. The results remained materially unchanged (Supplementary Table S7).

## 4. Discussion

This study demonstrates that higher SDH scores were associated with a lower likelihood of VI among Chinese adults aged ≥ 45 years. Furthermore, among those with VI, higher SDH scores were also correlated with a decreased risk of premature death. These associations remained robust after adjusting for confounding factors, were consistent across all subgroups analyzed, and were further validated by additional sensitivity analyses.

Consistent with earlier findings, our study shows an inverse association between SDH scores and VI risk. A study from the United States reported a strong link between VI and dementia in older adults [16]. Similarly, a UK study showed that visual search function was impaired in individuals with specific cognitive impairment [17]. These observations link cognitive decline to VI, while better cognitive function is associated with favorable SDH indicators such as social engagement and quality of life [18]. Evidence from a Chinese longitudinal study indicates that older adults with advanced age, lower educational attainment, and limited rural transportation access are at increased risk of VI [19]. Low socioeconomic status may increase the risk of VI through multiple pathways, including poor dietary patterns, accelerated aging, as well as barriers related to the accessibility, affordability, and utilization of eye care services, such as transportation difficulties, financial burden, inadequate insurance coverage, and limited eye health literacy [20,21]. Likewise, declining personal income has been linked to worsening visual function in a U.S. study [22]. Taken together, accumulating evidence from multiple populations consistently demonstrates that adverse SDH factors are independent predictors of higher VI risk.

Prior work has established a link between SDH and premature death. A cross-sectional study found that unemployment, lack of health insurance, lack of a steady partner, low educational level, and low household income were all significantly tied to premature death [12]. These same SDH factors have been tied to chronic conditions such as diabetes and cardiovascular disease, which in turn reduce life expectancy [23,24]. Our study further confirms these findings, showing that in adults with VI aged ≥ 45 years, the lower the SDH scores, the higher the risk of premature death.

Biological and behavioral factors likely interact to explain the observed associations of SDH with VI and with premature death among those with VI. At the physiological level, adverse SDH conditions can contribute to psychological distress and activate the sympathetic nervous system, thereby raising cardiovascular and metabolic risk, which in turn may predispose individuals to VI [25]. Behaviorally, individuals with lower SDH scores tend to have weaker health awareness and limited access to medical resources, thereby reducing their ability to manage their own health. Once VI occurs, daily living difficulties often lead to reduced physical activity, speeding up age-related decline [26]. VI also restricts social interaction, leading to loneliness and anxiety that harm health [27]. These forces can trigger a feedback loop in which social disadvantage and poor health reinforce one another.

This study has several key strengths. First, its reliance on the nationwide, large-sample CHARLS database supports the generalizability of the results. Second, we adjusted for numerous factors, including demographic, behavioral, and disease burden variables. Third, multiple sensitivity analyses consistently confirmed the stability of our findings. Finally, this study identifies a potential vicious cycle: social disadvantage leads to VI, which further worsens social disadvantage, ultimately resulting in premature death.

Several limitations of this study warrant consideration. First, VI was based on self-report, not clinical examination, which may have introduced recall bias. While self-report has its utility in large cohort studies, it does not equate to formal ophthalmic testing. However, these CHARLS measures have been validated against objective tests and used previously; any misclassification would likely attenuate rather than inflate our findings [28,29,30,31]. Second, the SDH indicators were relatively limited and did not include other factors such as food security. Third, the SDH composite score assigned equal weights to all 10 components, whereas different social determinants may exert differential effects on health outcomes. The use of an equal-weight index precluded us from determining whether individual indicators, such as geographic accessibility to eye care, financial affordability of treatment, or health insurance coverage, contributed differently to the risk of VI and premature mortality. Future studies employing refined weighting approaches or disaggregated analyses are warranted to further elucidate the relative importance of these factors for VI and premature death. Fourth, the study only covered individuals aged 45 and older, so the results cannot be generalized to younger age groups. Fifth, as with most longitudinal cohort studies, missing data led to the exclusion of some participants from the analytical sample. However, we performed multiple imputation for covariates with missing rates below 20% in the main analysis and conducted complete-case analyses using the original data in sensitivity analyses. The consistency of results across these approaches suggests that selection bias due to missing data is unlikely to have substantially influenced our findings. Sixth, SDHs and all covariates were assessed only at baseline. Given the dynamic nature of these factors over the 7-year follow-up period, temporal variations in SDH and health-related covariates could not be accounted for, which may have influenced the observed associations. Future studies employing repeated SDH measurements and time-varying covariate analyses are warranted to further validate these findings. Finally, the relatively short follow-up duration may have limited the number of mortality events. Future research involving multiple cohorts could help assess the generalizability to other populations of these findings. Furthermore, the role of inflammatory pathways could be explored.

## 5. Conclusions

In summary, higher SDH scores were associated with a lower risk of VI and premature death among Chinese adults aged ≥ 45 years. Among those with VI, lower social status was significantly associated with premature death. This evidence emphasizes an important role of prioritizing health equity in eye health and geriatric care policies, and implementing comprehensive social interventions aimed at reducing health inequalities associated with visual disability.

## Figures and Tables

**Table 1:** Baseline characteristics of participants without visual impairment (for VI incidence analysis, *n* = 2559).

Variable	Total (*n* = 2559)	Social Determinants of Health (<7) (*n* = 558)	Social Determinants of Health (≥7) (*n* = 2001)	Statistic	*p*
Age (SD), years	56.99 ± 8.97	59.92 ± 9.81	56.17 ± 8.55	T = 8.88	<0.001
Sex, *n* (%)				χ^2^ = 39.96	<0.001
Female	1202 (46.97)	328 (58.78)	874 (43.68)		
Male	1357 (53.03)	230 (41.22)	1127 (56.32)		
BMI (SD), Kg/m^2^	23.92 ± 3.50	23.39 ± 3.54	24.07 ± 3.48	T = –4.03	<0.001
Smoking status, *n* (%)				χ^2^ = 8.77	0.012
Never	1514 (59.16)	360 (64.52)	1154 (57.67)		
Former	201 (7.85)	41 (7.35)	160 (8.00)		
Current	844 (4.26)	157 (28.41)	687 (34.33)		
Drinking status, *n* (%)				χ^2^ = 33.31	< 0.001
Never	1451 (56.70)	365 (65.41)	1086 (54.27)		
Former	183 (7.15)	49 (8.78)	134 (6.70)		
Current	925 (36.15)	144 (25.81)	781 (39.03)		
Heart disease, *n* (%)				χ^2^ = 3.98	0.046
No	2321 (90.70)	494 (88.53)	1827 (91.30)		
Yes	238 (9.30)	64 (11.47)	174 (8.70)		
Stroke, *n* (%)				χ^2^ = 3.96	0.047
No	2515 (98.28)	543 (97.31)	1972 (98.55)		
Yes	44 (1.72)	15 (2.69)	29 (1.45)		
Diabetes, *n* (%)				χ^2^ = 1.28	0.258
No	2450 (95.74)	539 (96.59)	1911 (95.50)		
Yes	109 (4.26)	19 (3.41)	90 (4.50)		
Hypertension, *n* (%)				χ^2^ = 2.19	0.139
No	2025 (79.13)	429 (76.88)	1596 (79.76)		
Yes	534 (20.87)	129 (23.12)	405 (20.24)		
Dyslipidemia, *n* (%)				χ^2^ = 2.57	0.109
No	2352 (91.91)	522 (93.55)	1830 (91.45)		
Yes	207 (8.09)	36 (6.45)	171 (8.55)		

Data are mean ± SD for continuous variables and *n* (%) for categorical variables. Group comparisons: *t*-tests (continuous) and chi-square tests (categorical). BMI, body mass index.

**Table 2:** Baseline characteristics of participants with visual impairment (for premature mortality analysis, *n* = 8612).

Variable	Total (*n* = 8612)	Social Determinants of Health (<7) (*n* = 2813)	Social Determinants of Health (≥7) (*n* = 5779)	Statistic	*p*
Age (SD), years	56.72 ± 6.59	58.10 ± 6.47	56.06 ± 6.54	T = 13.62	<0.001
Sex, *n* (%)				χ^2^ = 48.47	<0.001
Female	4706 (54.64)	1688 (60.01)	3018 (52.04)		
Male	3906 (45.36)	1125 (39.99)	2781 (47.96)		
BMI (SD), Kg/m^2^	23.72 ± 3.65	23.35 ± 3.76	23.90 ± 3.58	T = –6.50	<0.001
Smoking status, *n* (%)				χ^2^ = 12.45	0.002
Never	5282 (61.33)	1800 (63.99)	3482 (60.04)		
Former	681 (7.91)	209 (7.43)	472 (8.14)		
Current	2649 (30.76)	804 (28.58)	1845 (31.82)		
Drinking status, *n* (%)				χ^2^ = 51.76	<0.001
Never	5076 (58.94)	1734 (61.64)	3342 (57.63)		
Former	685 (7.95)	278 (9.88)	407 (7.02)		
Current	2851 (33.10)	801 (28.47)	2050 (35.35)		
Heart disease, *n* (%)				χ^2^ = 26.66	<0.001
No	7583 (88.05)	2404 (85.46)	5179 (89.31)		
Yes	1029 (11.95)	409 (14.54)	620 (10.69)		
Stroke, *n* (%)				χ^2^ = 15.22	<0.001
No	8422 (97.79)	2726 (96.91)	5696 (98.22)		
Yes	190 (2.21)	87 (3.09)	103 (1.78)		
Diabetes, *n* (%)				χ^2^ = 2.72	0.099
No	8045 (93.42)	2610 (92.78)	5435 (93.72)		
Yes	567 (6.58)	203 (7.22)	364 (6.28)		
Hypertension, *n* (%)				χ^2^ = 9.03	0.003
No	6405 (74.37)	2035 (72.34)	4370 (75.36)		
Yes	2207 (25.63)	778 (27.66)	1429 (24.64)		
Dyslipidemia, *n* (%)				χ^2^ = 1.48	0.224
No	7705 (89.47)	2533 (90.05)	5172 (89.19)		
Yes	907 (10.53)	280 (9.95)	627 (10.81)		

Data are mean ± SD for continuous variables and *n* (%) for categorical variables. Group comparisons: *t*-tests (continuous) and chi-square tests (categorical). BMI, body mass index.

**Table 3:** SDH scores: Associations with visual impairment (general adults) and premature death (individuals with visual impairment).

Variable	Model 1		Model 2		Model 3		Model 4	
	HR (95% CI)	*p*	HR (95% CI)	*p*	HR (95% CI)	*p*	HR (95% CI)	*p*
Study A								
SDH scores (Continuous)	0.93 (0.90~0.96)	<0.001	0.94 (0.90~0.97)	0.001	0.94 (0.91~0.98)	0.002	0.94 (0.91~0.98)	0.002
SDH scores (Category)								
SDH scores (<7)	Reference		Reference		Reference		Reference	
SDH scores (≥7)	0.82 (0.75~0.91)	<0.001	0.85 (0.77~0.94)	0.001	0.86 (0.78~0.95)	0.003	0.86 (0.78~0.95)	0.003
Study B								
SDH scores (Continuous)	0.80 (0.73~0.87)	<0.001	0.83 (0.76~0.90)	<0.001	0.84 (0.77~0.92)	<0.001	0.85 (0.78~0.93)	<0.001
SDH scores (Category)								
SDH scores (<7)	Reference		Reference		Reference		Reference	
SDH scores (≥7)	0.63 (0.52~0.76)	<0.001	0.66 (0.55~0.80)	<0.001	0.68 (0.56~0.82)	<0.001	0.71 (0.59~0.86)	<0.001

HR: Hazard ratio. CI: Confidence interval. SDH: Social determinants of health. VI: Visual impairment. Study A examined the association between SDH scores and VI risk in the general population. Study B assessed the relationship between SDH scores and premature death risk in individuals with VI. Model 1: Unadjusted. Model 2: Adjusted for age and sex. Model 3: Further adjusted for smoking status, drinking status, and BMI. Model 4: Additionally adjusted for hypertension, diabetes, stroke, heart disease, and dyslipidemia.

## Data Availability

The data we used come from a public source: the CHARLS survey. It can be found online at https://charls.pku.edu.cn/ (accessed on 4 February 2026). Our study did not create any new data. If other researchers need our analysis code or the specific data extracts we worked with, they can ask the corresponding author. We will share these materials when the request is reasonable.

## Supplementary Materials

The following supporting information can be downloaded at: https://www.mdpi.com/article/10.3390/healthcare14152329/s1: Table S1: Components and scoring of the social determinants of health index; Table S2: Distribution of missing data; Table S3: Variance inflation factor; Table S4: Schoenfeld residuals test of the proportional hazards assumption; Table S5: SDH scores: Associations with Visual Impairment (General Adults) and Premature Death (Individuals with Visual Impairment)—based on pre-imputation data; Table S6: SDH scores: Associations with visual impairment (general adults) and premature death (individuals with visual impairment)— Model 4 (further adjusted for pension insurance, health insurance, education level); Table S7: SDH scores: Associations with premature death (individuals with visual impairment)—excluding deaths within the first two years. Table S8: Absolute risks and risk differences for visual impairment and premature death by SDH Category. Figure S1: Subgroup analyses of social determinants of health (SDH) scores.

## Abbreviations

The following abbreviations are used in this manuscript:

CHARLS	China Health and Retirement Longitudinal Study
VI	vision impairment
SDH	social determinants of health
WHO	world health organization
RCS	restricted cubic spline
HR	hazard ratio
CI	confidence interval
BMI	body mass index
SD	standard deviation
