# Supplementary material for: Associations of Social Determinants of Health Scores with Visual Impairment and Premature Death in Visually Impaired Adults: Insights from CHARLS—A Cohort Study
Source: Healthcare (Basel). 2026 Aug 1;14(15):2329. doi: 10.3390/healthcare14152329 (PMC13466124; doi:10.3390/healthcare14152329)
Supplement: Supplementary file 1 [file healthcare-14-02329-s001.zip › healthcare-4343247-supplementary.pdf]

Supplementary Table S1 Components and scoring of the social determinants of health index.

Social determinants of health	Description	Score
**House ownership**	Is the house you currently reside in rented? If yes, it indicates that the house is being rented; if no, it suggests the house is owned.	0: points for renting;<br>1: point for owning the house.
**Household income**	The average household income per member was 9,000 RMB, derived by summing total earnings from wages, agricultural activities, and private enterprise operations, and dividing this sum by the number of individuals residing in the household.	0: Less than 9000 RMB；<br>1: Average total household income before tax exceeds 9000 RMB
**Employment status**	Have you ever worked? Are you retired?	0: No employment history or currently unemployed；<br>1: Retired or currently employed.
**Health Insurance**	(1) Urban employee medical insurance (yi-bao).<br>(2) Urban resident medical insurance.<br>(3) New cooperative medical insurance (he-zuo-yi-liao).<br>(4) Urban and rural resident medical insurance.<br>(5) Government medical insurance (gong-fei).<br>(6) Medical aid.<br>(7) Private medical Insurance: Purchased by R's union.<br>(8) Private medical Insurance: Purchased by Individual.<br>(9) Other medical insurance.<br>(10) No insurance.	0: Without health insurance or receiving government assistance;<br>1: Covered by government-funded insurance, private insurance, or self-purchased health plans.
**Educational Attainment**	Based on self-reported highest educational attainment.	0: Less than a bachelor's degree;<br>1: Bachelor's degree or higher"
**Access to Hospital Services**	The participants reported that transportation barriers prevented them from accessing medical care.	0: Transportation barriers<br>1: No transportation barriers, with medical facilities in proximity.
**Living alone**	Do you currently live alone?	0: Living Alone; 1: Co-living With

Supplementary Table S1 Components and scoring of the social determinants of health index.

Social determinants of health	Description	Score
	Yes/No	Others"
**Depression**	The CES-D score refers to the Center for Epidemiologic Studies Depression Scale (CES-D), a tool designed to assess the severity of depressive symptoms.	0: CES-D score exceeds 10;<br>1: Does not exceed 10.
**Social Activities**	In the past month, how often have you engaged in these activities (volunteer or charitable activities, caring for patients or disabled individuals, helping family members, friends, or neighbors, attending educational or training courses, socializing with friends, engaging in sports or recreational activities, participating in social or other types of clubs, attending organizational activities, trading stocks, or browsing the internet)? Was it nearly every day, nearly every week, or not frequently?	0: All activities are 'not frequent';<br>1: Participates frequently in at least one activity.
**Marital Status**	What is your current marital status?	0: Separated, divorced, widowed, or never married；<br>1: married and cohabiting, or in a Long-term Partnership.

Supplementary Table S2 Distribution of missing data.

Characteristic	Study A		Study B	
	**No. of missing values**	**Percentage (%)**	**No. of missing values**	**Percentage (%)**
**Age**	0	0.00	0	0.00
**Sex**	0	0.00	0	0.00
**Drinking status**	0	0.00	1	0.01
**Smoking status**	0	0.00	2	0.02
**Body Mass Index**	417	16.30	1104	12.82
**Heart Disease**	14	0.55	48	0.56
**Diabetes**	14	0.55	91	1.06
**Hypertension**	9	0.35	48	0.56

SDH: Social determinants of health. Study A: Association of SDH scores with visual impairment in the general population. Study B: Association of SDH scores with premature death in individuals with visual impairment.

Supplementary Table S3 Variance inflation factor.

Characteristic	GVIF	Df	GVIF^(1/(2×Df))
Study A			
Social determinants of health	1.095	1	1.046
Age	1.151	1	1.073
Sex	1.961	1	1.400
BMI	1.135	1	1.065
Smoking status	1.749	2	1.150
Drinking status, n(%)	1.463	2	1.100
Heart disease, n(%)	1.077	1	1.038
Stroke, n(%)	1.028	1	1.014
Diabetes, n(%)	1.035	1	1.017
Hypertension, n(%)	1.186	1	1.089
Dyslipidemia, n(%)	1.113	1	1.055
Study B			
Social determinants of health	1.045	1	1.022
Age	1.081	1	1.040
Sex	2.101	1	1.450
BMI	1.216	1	1.103
Smoking status	2.043	2	1.196
Drinking status, n(%)	1.433	2	1.094
Heart disease, n(%)	1.096	1	1.047
Stroke, n(%)	1.063	1	1.031
Diabetes, n(%)	1.147	1	1.071
Hypertension, n(%)	1.239	1	1.113
Dyslipidemia, n(%)	1.207	1	1.098

SDH: Social determinants of health. Study A: Association of SDH scores with visual impairment in the general population. Study B: Association of SDH scores with premature death in individuals with visual impairment.

Supplementary Table S4 Schoenfeld residuals test of the proportional hazards assumption.

**Variables**	**Chi-sq**	**Df**	*p*
Study A			
Social determinants of health	0.997	1	0.318
Age	0.456	1	0.499
Sex	0.587	1	0.444
BMI	0.632	1	0.427
Smoking status	3.443	2	0.179
Drinking status, n(%)	0.047	2	0.977
Heart disease, n(%)	2.587	1	0.108
Stroke, n(%)	5.581	1	0.018
Diabetes, n(%)	2.232	1	0.135
Hypertension, n(%)	0.732	1	0.392
Dyslipidemia, n(%)	3.344	1	0.067
GLOBAL	22.014	13	0.055
Study B			
Social determinants of health	1.754	1	0.185
Age	0.013	1	0.908
Sex	0.312	1	0.576
BMI	0.238	1	0.401
Smoking status	1.827	2	0.075
Drinking status, n(%)	5.169	2	0.626
Heart disease, n(%)	<.001	1	0.983
Stroke, n(%)	0.045	1	0.831
Diabetes, n(%)	2.880	1	0.090
Hypertension, n(%)	0.315	1	0.574
Dyslipidemia, n(%)	3.277	1	0.070
GLOBAL	16.998	13	0.199

Study A: The association between social determinants of health scores and the risk of visual impairment among general population. Study B: The analysis of the association between social determinants of health scores and the risk of premature death among individuals with visual impairment.

Supplementary Table S5 SDH scores: Associations with Visual Impairment (General Adults) and Premature Death (Individuals with Visual Impairment)— based on pre-imputation data.

**Variable**	**Model1**		**Model2**		**Model3**		**Model4**	
	**HR (95%CI)**	***p***	**HR (95%CI)**	***p***	**HR (95%CI)**	***p***	**HR (95%CI)**	***p***
Study A								
SDH scores (Continuous)	0.93 (0.90 ~ 0.96)	<.001	0.94 (0.90 ~ 0.97)	0.001	0.95 (0.91 ~ 0.99)	0.016	0.95 (0.91 ~ 0.99)	0.016
SDH scores (Category)								
SDH scores (< 7)	Reference		Reference		Reference		Reference	
SDH scores (≥ 7)	0.82 (0.74 ~ 0.91)	<.001	0.85 (0.77 ~ 0.94)	0.001	0.87 (0.78 ~ 0.97)	0.016	0.87 (0.78 ~ 0.98)	0.020
Study B								
SDH scores (Continuous)	0.80 (0.73 ~ 0.87)	<.001	0.83 (0.76 ~ 0.90)	<.001	0.83 (0.76 ~ 0.92)	<.001	0.85 (0.77 ~ 0.94)	0.001
SDH scores (Category)								
SDH scores (< 7)	Reference		Reference		Reference		Reference	
SDH scores (≥ 7)	0.63 (0.52 ~ 0.76)	<.001	0.66 (0.55 ~ 0.80)	<.001	0.67 (0.55 ~ 0.83)	<.001	0.71 (0.58 ~ 0.87)	0.001

HR: Hazard Ratio. CI: Confidence Interval. SDH: Social determinants of health. VI: Visual impairment. Study A examined the association between SDH scores and VI risk in the general population. Study B assessed the relationship between SDH scores and premature death risk in individuals with VI. Model1: Unadjusted. Model2: adjusted for age and sex. Model3: further adjusted for smoking status, drinking status, and BMI. Model4: additionally adjusted for hypertension, diabetes, stroke, heart disease, and dyslipidemia.

Supplementary Table S6 SDH scores: Associations with Visual Impairment (General Adults) and Premature Death (Individuals with Visual Impairment)— Model 4 (further adjusted for pension insurance, health insurance, education level).

Variable	Model1		Model2		Model3		Model4	
	**HR (95%CI)**	***p***	**HR (95%CI)**	***p***	**HR (95%CI)**	***p***	**HR (95%CI)**	***p***
Study A								
SDH scores (Continuous)	0.93 (0.90 ~ 0.96)	<.001	0.94 (0.90 ~ 0.97)	<.001	0.94 (0.91 ~ 0.98)	0.002	0.94 (0.90 ~ 0.98)	0.002
SDH scores (Category)								
SDH scores (< 7)	Reference		Reference		Reference		Reference	
SDH scores (≥ 7)	0.82 (0.74 ~ 0.91)	<.001	0.85 (0.77 ~ 0.94)	0.001	0.86 (0.78 ~ 0.95)	0.003	0.86 (0.78 ~ 0.95)	0.003
Study B								
SDH scores (Continuous)	0.80 (0.73 ~ 0.87)	<.001	0.83 (0.76 ~ 0.90)	<.001	0.84 (0.77 ~ 0.92)	<.001	0.85 (0.78 ~ 0.94)	0.001
SDH scores (Category)								
SDH scores (< 7)	Reference		Reference		Reference		Reference	
SDH scores (≥ 7)	0.63 (0.52 ~ 0.76)	<.001	0.66 (0.55 ~ 0.80)	<.001	0.68 (0.56 ~ 0.82)	<.001	0.72 (0.59 ~ 0.88)	0.001

HR: Hazard Ratio. CI: Confidence Interval. SDH: Social determinants of health. VI: Visual impairment. Study A examined the association between SDH scores and VI risk in the general population. Study B assessed the relationship between SDH scores and premature death risk in individuals with VI. Model1: Unadjusted. Model2: adjusted for age and sex. Model3: further adjusted for smoking status, drinking status, and BMI. Model4: additionally adjusted for hypertension, diabetes, stroke, heart disease, and dyslipidemia, pension insurance, medical insurance, education level.

Supplementary Table S7 SDH scores: Associations with Premature Death (Individuals with Visual Impairment)—excluding deaths within the first two years.

Variable	Model1		Model2		Model3		Model4	
	**HR (95%CI)**	***p***	**HR (95%CI)**	***p***	**HR (95%CI)**	***p***	**HR (95%CI)**	***p***
SDH scores (Continuous)	0.84 (0.75 ~ 0.92)	<.001	0.86 (0.78 ~ 0.96)	0.006	0.88（0.79 ~ 0.98）	0.016	0.89（0.80 ~ 0.99）	0.035
SDH scores (Category)								
SDH scores (< 7)	Reference		Reference		Reference		Reference	
SDH scores (≥ 7)	0.69 (0.55 ~ 0.86)	0.001	0.72 (0.57 ~ 0.90)	0.004	0.74 (0.59 ~ 0.93)	0.009	0.78 (0.62 ~ 0.98)	0.031

HR: Hazard Ratio. CI: Confidence Interval. SDH: Social determinants of health. VI: Visual impairment. Study A examined the association between SDH scores and VI risk in the general population. Model1: Unadjusted. Model2: adjusted for age and sex. Model3: further adjusted for smoking status, drinking status, and BMI. Model4: additionally adjusted for hypertension, diabetes, stroke, heart disease, and dyslipidemia.

Supplementary Table S8 Absolute Risks and Risk Differences for Visual Impairment and Premature Death by SDH Category.

Outcome	SDH < 7	SDH ≥ 7	Absolute Risk Difference (95%CI)	HR (95% CI) (from Table 3)
Study A				
Event rate, % (n/N)	92.1% (514/558)	86.9% (1740/2001)	5.2 (2.5 ~ 7.8)	0.86 (0.78 ~ 0.95)
Study B				
Event rate, % (n/N)	6.8% (191/2813)	4.5% (262/5779)	2.3 (1.2 ~ 3.3)	0.71 (0.59 ~ 0.86)

SDH: Social determinants of health. VI: Visual impairment. Study A examined the association between SDH scores and VI risk in the general population. Study B assessed the relationship between SDH scores and premature death risk in individuals with VI. HR values are from the fully adjusted model (Model 4) in Table 3, comparing SDH ≥7 vs. SDH <7.

Supplementary Figure S1. Subgroup analyses of social determinants of health scores.

A

B

C

D

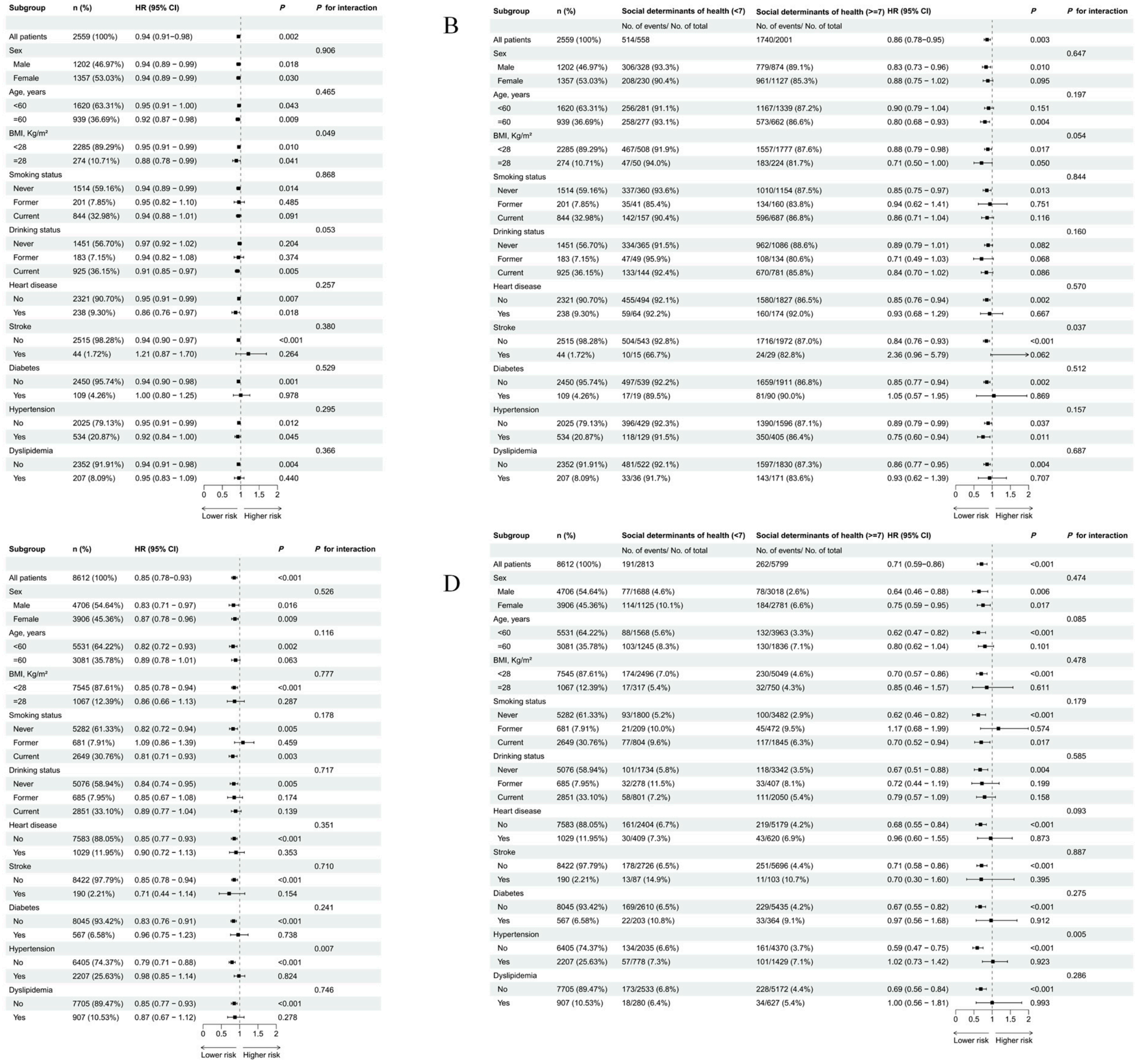

Supplementary Figure S1 A and B show the association between social determinants of health scores and visual impairment among general adults. Supplementary Figure S1 C and D show the association with premature death among individuals with visual impairment. Social determinants of health scores were modeled as: continuous in Supplementary Figure S1 A and C; categorical (≥7 vs. <7) in Supplementary Figure S1 B and D.
